# Management of Perforated Appendicitis in Amyand's Hernia: A Multidisciplinary Approach to Complex Postoperative Complications

**DOI:** 10.7759/cureus.81498

**Published:** 2025-03-31

**Authors:** Laura Miranda Burgos, Alphonsa Thomas, Wayne Fluss, Aryama D Sharma

**Affiliations:** 1 Internal Medicine, Broward Health North, Deerfield Beach, USA; 2 Gastroenterology, Broward Health North, Deerfield Beach, USA

**Keywords:** acute appendicitis, amyand's hernia, hernia repair, intra-abdominal abscess, intra-abdominal infection, perforated appendicitis, psoas muscle abscess, purulent peritonitis, scrotal abscess, strangulated inguinal hernia

## Abstract

Amyand's hernia is an exceptionally rare condition in which the appendix is located within an inguinal hernia sac. While it is typically asymptomatic, the occurrence of appendicitis or perforation within these hernias presents significant diagnostic and therapeutic challenges. We report a unique case involving a 31-year-old male patient who developed perforated appendicitis within an Amyand's hernia, resulting in severe intra-abdominal infection and complex postoperative complications, including the formation of multiple abscesses at three different sites. A multidisciplinary approach, involving general surgery, infectious disease, interventional radiology (IR), and urology, was crucial for effective source control and infection management. This case underscores the importance of individualized surgical decision-making in cases of Amyand's hernia with significant inflammatory involvement, emphasizing the necessity for timely recognition and intervention to minimize complications and improve patient outcomes.

## Introduction

Amyand's hernia is a rare condition characterized by the presence of the appendix within an inguinal hernia sac. It was first described by Claudius Amyand in 1735 and has an estimated prevalence of less than 1% among all inguinal hernias [1,2]. Even rarer is the manifestation of appendicitis within this type of hernia, which accounts for about 0.1% of all appendicitis cases [3]. Amyand's hernias are typically asymptomatic, with the majority of cases presenting as incidental findings during surgery for presumed incarcerated or strangulated inguinal hernias [4]. The occurrence of perforated appendicitis within an Amyand's hernia is also uncommon, representing approximately 16.1% of Amyand's hernia cases that involve appendicitis [5]. Early diagnosis and management are critical to prevent serious complications, such as intra-abdominal infections, abscess formation, surgical site infections, hernia recurrence, and prolonged recovery [6]. In this report, we describe a case involving a 31-year-old male patient with perforated appendicitis within an Amyand's hernia. We highlight the patient's clinical course, the surgical decision-making process, and the importance of interdisciplinary collaboration in managing postoperative complications.

## Case presentation

A 31-year-old male patient with a known history of an uncomplicated right inguinal hernia presents to the emergency department with a one-day history of excruciating right lower quadrant abdominal pain. He described the pain as severe, gradual, constant, localized, and dull-like, associated with right groin pain and swelling, decreased appetite, nausea, and a few episodes of non-bloody, non-bilious emesis. He denied fever, chills, changes in weight, changes in bowel habits, melena, or hematochezia. He denied any recent injury or trauma. His past medical history was significant for tobacco use disorder, polysubstance use disorder, and alcohol use disorder. He denied any prior surgical history. Upon arrival, vital signs were remarkable for tachycardia, with a heart rate of 104 beats per minute. Physical examination revealed a distended abdomen with tenderness to light palpation in the right lower quadrant, guarding, rebound tenderness, and a painful, right-sided inguinal hernia that was non-reducible at the time. Laboratory studies were notable for an elevated white blood cell count of 11.44 × 10^3^ cells/µL with neutrophilia of 90.8%, alanine aminotransferase level of 76 U/L, aspartate aminotransferase level of 97 U/L, serum total bilirubin level of 3.8 mg/dL, direct bilirubin level of 0.2 mg/dL, alkaline phosphatase level of 135 U/L, lipase level of 15 U/L, and a lactic acid level of 1.5 mmol/L (Table 1).

**Table 1:** Laboratory analysis of the patient in reference to normal range

Parameter	Result	Reference range
White blood cells	11.44 × 10^3^ cells/µL	4.5-11.0 × 10^3^ cells/μL
Neutrophils	90.8%	40%-60%
Alanine aminotransferase	76 U/L	7-56 U/L
Aspartate aminotransferase	97 U/L	8-40 U/L
Total bilirubin	3.8 mg/dL	0.1-1.2 mg/dL
Direct bilirubin	0.2 mg/dL	0.0-0.3 mg/dL
Alkaline phosphatase	135 U/L	44-120 U/L
Lipase	15 U/L	10-140 U/L
Lactic acid	1.5 mmol/L	0.5-2.2 mmol/L

**Figure 1:**
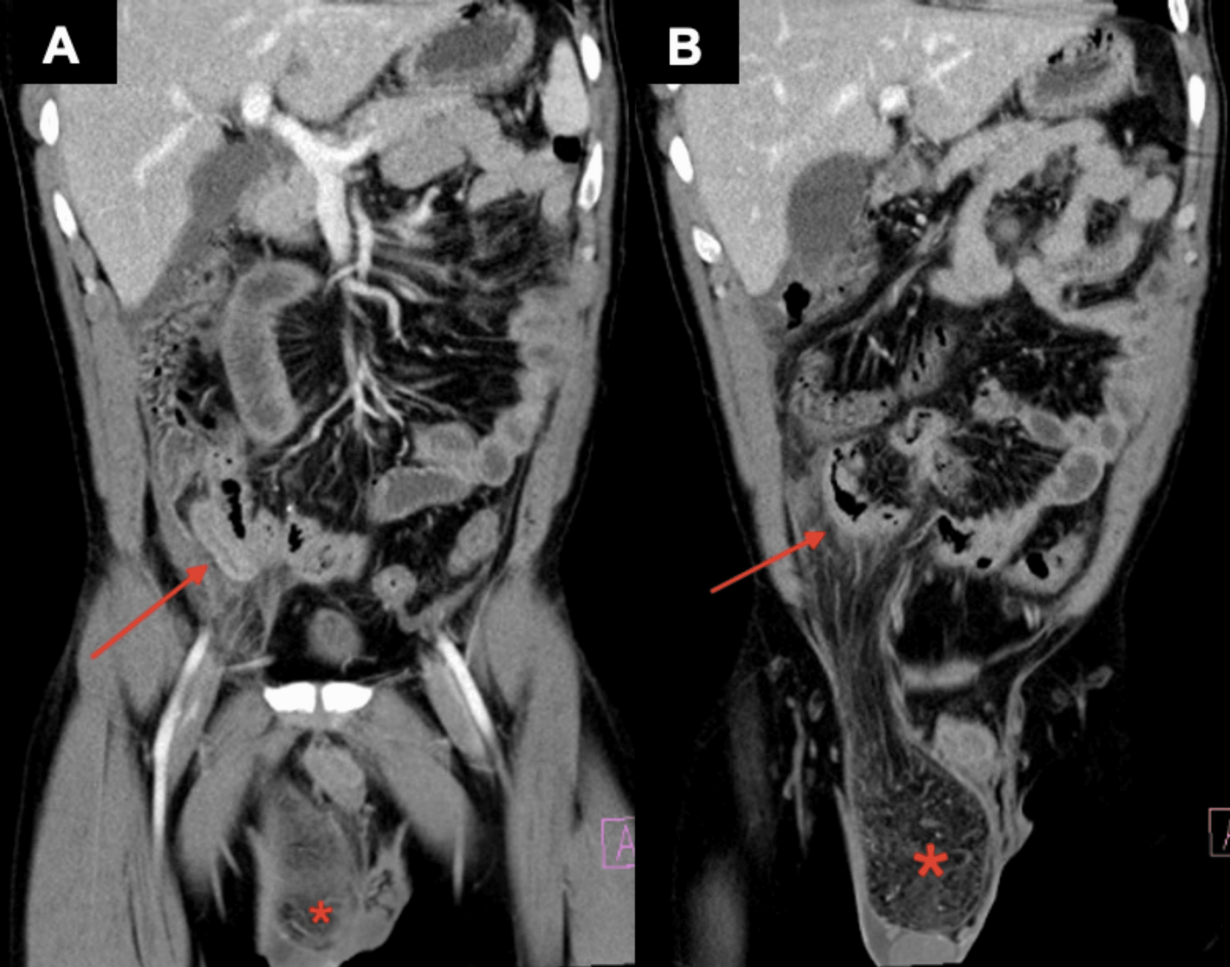
Coronal (A and B) CT images of the abdomen and pelvis with intravenous contrast demonstrating a right-sided Amyand's hernia with perforated appendicitis There is radiographic evidence of an inflamed appendix (red arrow) protruding into the hernia sac (red asterisk). CT: computed tomography

**Figure 2:**
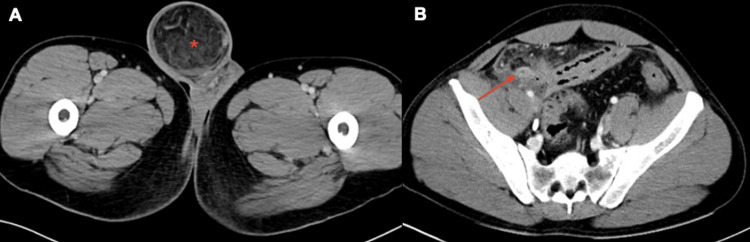
Axial (A and B) images of the abdomen and pelvis with intravenous contrast demonstrating a right-sided Amyand's hernia with perforated appendicitis There is radiographic evidence of an inflamed appendix (red arrow) protruding into the hernia sac (red asterisk). CT: computed tomography

A computed tomography (CT) of the abdomen and pelvis demonstrated a right inguinal hernia containing fat and an enlarged, inflamed appendix protruding into the hernia sac with evidence of extraluminal air, small volume ascites, and peritoneal inflammatory changes with diffuse bowel thickening (Figure 1 and Figure 2).

**Figure 3:**
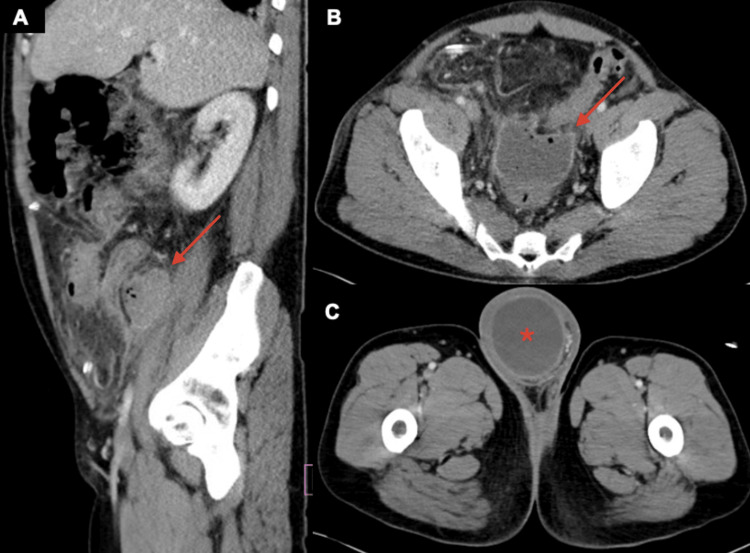
Sagittal (A) and axial (B and C) CT images of the abdomen and pelvis with oral and intravenous contrast demonstrating a 3.5 x 4.9 cm right psoas muscle abscess (A, red arrow), a 4.4 x 6.6 cm deep pelvic abscess (B, red arrow), and fluid extending into the scrotum via a patent inguinal canal, suggestive of abscess versus reactive hydrocele (C, red asterisk) CT: computed tomography

The patient was started on intravenous piperacillin-tazobactam for empiric antibiotic coverage, and general surgery was consulted. He underwent a laparoscopic appendectomy, which revealed purulent peritonitis and a necrotic appendix protruding into the inguinal hernia sac with significant surrounding purulence. The omentum within the hernia sac was intact and easily reduced into the abdomen. Given the extensive purulence and inflammation associated with this patient's ruptured appendix, a decision was made to place a Jackson-Pratt (JP) drain into the right lower quadrant and defer his hernia repair to a later time following a staged approach. On postoperative day 7, the patient developed marked diffuse abdominal pain and distension and worsening leukocytosis of 18.84 × 10^3^ cells/µL. Blood cultures were negative. A repeat CT of the abdomen and pelvis with oral intravenous and intravenous contrast showed a 3.5 x 4.9 cm abscess anterior to the right psoas muscle, a 4.4 x 6.6 cm deep pelvic abscess within the lower pelvis anterior to the rectum, and fluid extending into the scrotum via a patent inguinal canal (Figure 3).

Meropenem and daptomycin were initiated for broad-spectrum antimicrobial coverage, with micafungin added for antifungal therapy. Interventional radiology (IR) and urology were consulted for the management of these fluid collections, which were consistent with postoperative abscesses. The patient underwent a percutaneous CT-guided drainage of all three fluid collections followed by a scrotal surgical exploration with evacuation of fluid collection and Penrose catheter insertion into the right hemiscrotum, facilitating resolution of the abscesses and adequate source control.

## Discussion

Amyand's hernia is a rare clinical entity in which the appendix is located within an inguinal hernia sac, with an even rarer subset presenting with acute appendicitis or perforation [7]. The presence of perforated appendicitis within an Amyand's hernia poses both diagnostic and therapeutic challenges, as its symptoms can mimic those of incarcerated or strangulated hernias, often leading to delays in recognition and intervention [8]. Literature indicates that the most common presenting symptom of an Amyand's hernia is a painful inguinal or inguinoscrotal swelling, occurring in about 83% of cases [9]. While the pathophysiology is not fully understood, it is believed that chronic mechanical compression of the appendix within the hernia sac may hinder lymphatic and venous drainage, resulting in inflammation and eventual perforation [10].

This case highlights the complexity of managing Amyand's hernia with perforated appendicitis and its related complications. Simple cases of Amyand's hernia with a non-inflamed appendix can typically be managed with elective hernia repair. However, cases involving appendicitis or perforation require a more individualized approach [11]. Laparoscopic appendectomy has become increasingly preferred over open techniques when managing appendicitis, as it allows for thorough exploration of the peritoneal cavity and reduces postoperative morbidity [12]. In this case, due to extensive purulence and peritoneal involvement, primary hernia repair was deferred in favor of a staged approach to minimize the risk of surgical site infection and recurrence. Generally, in cases of perforated appendicitis or significant contamination, immediate hernia repair, especially using mesh, is avoided to reduce the risk of infection and other complications. Instead, a staged approach is preferred, prioritizing appendectomy and infection management, with hernia repair deferred until the inflammation has subsided [13]. Studies by Papaconstantinou et al. [5] and Ranganathan et al. [14] emphasize the importance of tailoring hernia repair to individual patients, suggesting that deferring mesh repair in cases of significant contamination leads to improved outcomes.

A systematic review of acute cases of Amyand's hernia reported a mortality rate of 1.8% among patients with complicated presentations, including perforated appendicitis. The morbidity rate for these cases is also significant, reported at 9.2%, including complications such as surgical site infections (3.6%) and other postoperative issues [15]. A particularly notable aspect of this case was the development of multiple postoperative abscesses at three distinct locations: the right psoas muscle, the lower pelvis anterior to the rectum, and the right hemiscrotum via a patent inguinal canal. The tracking of fluid through the inguinal canal into the scrotum highlights the potential for deep pelvic infections to extend along anatomical planes, complicating postoperative recovery. These complications underscore the importance of close postoperative monitoring, early imaging in the event of clinical deterioration, and prompt multidisciplinary intervention. Our approach to managing these complications involved broad-spectrum antimicrobial therapy alongside an interdisciplinary effort that included general surgery, infectious disease, interventional radiology, and urology. The decision to proceed with percutaneous drainage of the psoas and pelvic abscesses, along with scrotal surgical exploration, proved effective in achieving source control. The addition of antifungal therapy was based on the patient's persistent inflammatory response and clinical suspicion of fungal superinfection in the setting of prolonged antibiotic therapy.

## Conclusions

Amyand's hernia with perforated appendicitis is a rare but serious condition requiring prompt diagnosis and tailored management. This case highlights the critical role of a multidisciplinary approach in managing complex postoperative complications, including abscess formation in multiple anatomical compartments. Early recognition, appropriate surgical intervention, and close postoperative monitoring are crucial to optimizing patient outcomes. Staged surgical management, along with the use of percutaneous drainage techniques and targeted antimicrobial therapy, can effectively reduce morbidity and improve recovery in such challenging cases. Potential limitations to this approach include increased risk of infection, delayed hernia repair, higher patient morbidity, complexity of subsequent surgery, and greater resource utilization. Clinicians should remain vigilant for complications following surgical intervention and adopt a collaborative approach to address these challenges effectively.
